# Wireless Temperature, Relative Humidity and Occupancy Monitoring System for Investigating Overheating in Buildings

**DOI:** 10.3390/s22228638

**Published:** 2022-11-09

**Authors:** Dóra Szagri, Bálint Dobszay, Balázs Nagy, Zsuzsa Szalay

**Affiliations:** 1 Department of Construction Materials and Technologies, Faculty of Civil Engineering, Budapest University of Technology and Economics, Műegyetem rkp. 3, 1111 Budapest, Hungary; 2 Building Construction Design Kft., Harmat u. 20, 1105 Budapest, Hungary

**Keywords:** wireless sensor network, ESP32, user behaviour, motion detection, heatwave, summer overheating, temperature, ODH_26_, prefabricated reinforced concrete panel building

## Abstract

With the climate change we are experiencing today, the number and intensity of heatwaves are increasing dramatically, significantly impacting our buildings’ overheating. The majority of the prefabricated concrete panel buildings in Hungary are considered outdated from an energy point of view. These buildings may be at greater risk from extreme weather events. To examine this, long-term monitoring measurements are needed. Therefore, we developed a unique, reliable, and cost-effective wireless monitoring system, which can track in real time the indoor air quality data (temperature, relative humidity, CO_2_) of the investigated apartment building, as well as users’ habits, such as resident presence, window opening, and blind movement. The data were used to analyse and quantify the summer overheating of the dwelling and user habits. The measurements showed that the average temperature in all rooms was above 26 °C, and there were several occasions when the temperature exceeded 30 °C. Overheating in apartment buildings in summer is a significant problem that needs to be addressed. Further investigation of ventilation habits will help develop favourable ventilation strategies, and using these measurements in dynamic simulations will also help improve the models’ validity for further studies.

## 1. Introduction

According to the latest available data [1] of the Hungarian Central Statistical Office, the share of prefabricated reinforced concrete sandwich panel block housing [2] is approximately 13.3% in Hungary, i.e., there are more than half a million “panel” dwellings, with 1,150,000 inhabitants. The vast majority of these buildings only contain a 5–8 cm insulation layer in the reinforced concrete large panel system which is now significantly aged and deteriorated, has not undergone in-depth renovation [3], and is considered outdated from an energy performance point of view. One of the consequences of climate change is an increase in the frequency of extreme weather events [4,5], such as the intensity and frequency of summer heatwaves [6,7], which may pose an even greater risk to panel dwellings [8]. Additionally, a study [9] showed that 10-storey housing blocks made of prefabricated reinforced concrete panels are among the most endangered building types in terms of climate vulnerability.

Regarding vulnerability, the older age group (65+) is most at risk from heatwaves. The proportion of flats occupied only by older people is the highest in Budapest (25%) and around 20% in the rest of the country. Considering these data, it is essential to address this type of building, as there are few real-time measurement data available.

The measurement of buildings’ indoor environment is usually performed using low-cost wireless monitoring devices capable of monitoring various parameters [10]. However, the limitations and capabilities are worth keeping an eye on if anyone uses low-cost sensors, since many cheap sensors can produce significant errors [11]. The most studied indoor air parameters in wireless low-cost IoT devices are temperature and relative humidity [12,13,14,15,16,17,18]. Monitoring the carbon dioxide concentration of the indoor air with low-cost sensors is also used to evaluate indoor air quality, occupancy, or building performance [19,20,21,22]. For detecting motion and indoor presence, passive infrared sensor (PIR) arrays can be placed in rooms or doorways [23], or magnetic reed switches can be used to act as door sensors [24]. Window opening is usually measured using magnetic switches [25,26,27]. Since shading plays a significant role in creating an adequate state of comfort in the interior in both winter and summer [25], monitoring of the window shading devices (e.g., blinds, shutters) can also be essential, and magnetic sensors could be a suitable solution [26].

We aimed to develop a wireless sensor network to measure the indoor air quality of a panel dwelling in Budapest. This system must be affordable and reliable, and an interactive online interface is also required to track measurements and monitor user habits. The monitoring measurements will provide a realistic picture of the summer temperature and relative humidity in the dwelling, as well as the evolution of the occupant’s habits (presence, ventilation, and shading).

## 2. Materials and Methods

### 2.1. Analysed Building and Apartment

**Figure 1:**
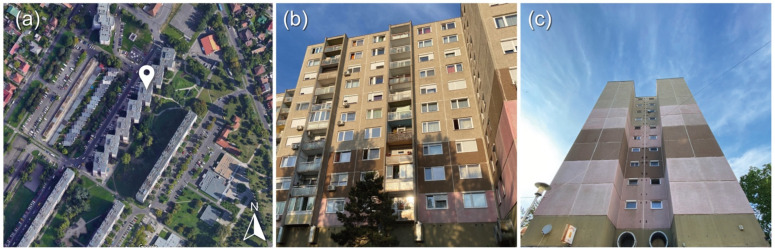
(**a**) Location of the building (source: Google Maps); (**b**) west façade of the building; (**c**) south façade of the building.

The analysed apartment is in the Havanna residential area, a part of Budapest in the XVIII district (see Figure 1). The Havanna residential area was built in two phases between 1977 and 1985 on almost 60 hectares of land. This part of the district has a 6248-unit housing estate built with prefabricated technology. Nearly a third of the district’s population lives in housing estates, and about 15% of the district’s population lives in the analysed area [27]. The number of people in the 55–64 age group is extremely high. The housing estates built in the late 1970s and early 1980s were occupied by many young people who all moved in at the same time, many of whom still live there today. The number of older people in this group is expected to increase. Many of the buildings are outdated, and the energy efficiency of the housing stock is low. In general, the thermal insulating performance of external walls, roofs, and fenestration is poor, the technology of mechanical systems and equipment is also outdated, and there is frequent overconsumption of district heating in the winter [27]. Considering the above, the chosen building is rather fragile; thus, detailed monitoring measurements could help facilitate further improvements.

The analysed apartment is located on the seventh floor of a 10-story panel building (Figure 1). The thermal properties of these structures are difficult to determine based on existing documents, as deterioration over time, manufacturing technology defects, and materials ageing all contribute to unfavourable and insecure values. The building is heated from several sides, and the thermal transmittance (U-value) of the boundary external wall system is around 0.9 W/m^2^K [28]. The doors and windows in the apartment are not original; they have been replaced by double-layer fenestration with plastic frames. External shutters are only applied in one room and the kitchen. There is also no air conditioning. In the dining room, there is one ceiling to increase air circulation in the summer. According to the residents, most of the time, it is not too hot in the apartment during the summer; however, some periods are difficult to bear.

**Figure 2:**
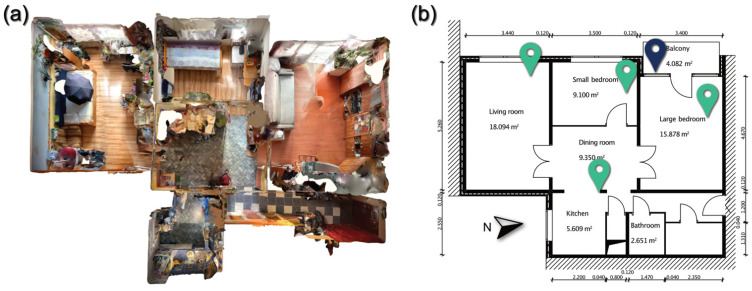
(**a**) LiDAR survey; (**b**) floor plan and layout of the studied dwelling with the indicated position of the room sensors.

Figure 2 shows the apartment’s layout and size and the sensors’ marked locations. During the preliminary survey, we took a coloured point cloud of the dwelling using a 2020 Apple iPad Pro’s (Apple Inc., USA) built-in LiDAR technology with the Polycam application [29], which allowed us to quickly survey the geometry of the flat and determine the dimensions needed to design the monitoring system without disturbing residents, with acceptable accuracy. Using the coloured point cloud from the LiDAR survey, we were not only able to draw the floor plan of the flat (see Figure 2), but we could locate the position of every piece of furniture, power outlets, and other moveable appliances in three dimensions, as the coloured point clouds can be used similarly to 360° pictures. Without colouring, the point cloud only contains the shapes of the surveyed objects, and it is much harder to identify the furniture and power outlets, which were helpful when we designed the location of the sensors. Besides the 2D floor plan and areas calculated according to the geometry represented in the floor plan, the room heights from vertical sections were also obtained from the LiDAR survey. Capturing coloured point clouds using the LiDAR built-in current Apple tablets and smartphones certainly has its limitations [30,31], even depending on the software used for capturing the point cloud [32], but in small-scale applications, it can be a great help. It can significantly speed up surveys by recording all the necessary information simultaneously compared to manual surveying.

The apartment is 69.71 m^2^ with a 4.08 m^2^ balcony. The hallway leads to the bathroom, toilet, and dining room. There are three bedrooms, one of which is used as an office and living room. During the measurements, four room sensors were placed in the small bedroom, the large bedroom, the junction of the kitchen and dining room, and the living room (see Figure 2). A sensor was also installed on the outside balcony to collect data on outside air temperature, humidity, and CO_2_ concentration.

### 2.2. Monitoring System Overview

The monitoring system was expected to be affordable, reliable, and have an online interface for live monitoring of the measurement. The measured values have multiple uses. The indoor air condition and quality data can be used to assess the thermal comfort of the dwelling in summer and winter; the analysis of these data and user habits will help to build a dynamic simulation, assess heating consumption, and draw conclusions about user habits for a given dwelling.

**Figure 3:**
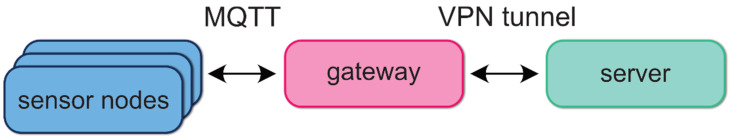
Overview of the main components.

The system consists of three main parts (Figure 3): the sensor nodes, the gateway, and the server. The sensor nodes use the MQTT protocol to send measurements to the server. The gateway encrypts the traffic using a VPN tunnel to the server. The server hosts the database and visualisation software to store and display measurements.

#### 2.2.1. Sensor Nodes

The sensor nodes are based on an ESP32-WROOM-32E (Espressif Systems, China) ESP32 module [33]. It is a low-cost microcontroller module with integrated Wi-Fi connectivity, low power consumption, and satisfactory software development support from the vendor (ESP-IDF framework). The nodes consist of the ESP32 module and the necessary sensors and power source, encapsulated in a custom 3D-printed case to minimise the device’s size.

#### 2.2.2. Room Measurement

The characterisation of the rooms consists of temperature, relative humidity, carbon-dioxide concentration measurements, and motion detection. The latter is supposed to indicate the human presence, affecting all the other measurements. Like the window nodes, these are also based on an ESP32 module. However, for these, the mains power is available, so it was not critical to design for battery operation; the nodes are powered using 5V wall adapters. 

The following chips are used for the measurements:

- Temperature and relative humidity: SHT85 (Sensirion AG, Switzerland) [34];
- CO_2_ concentration: SCD30 (Sensirion AG, Switzerland) [35]; and
- Motion detection: HC-SR501 PIR sensor (TruSens, China) [36].

**Figure 4:**
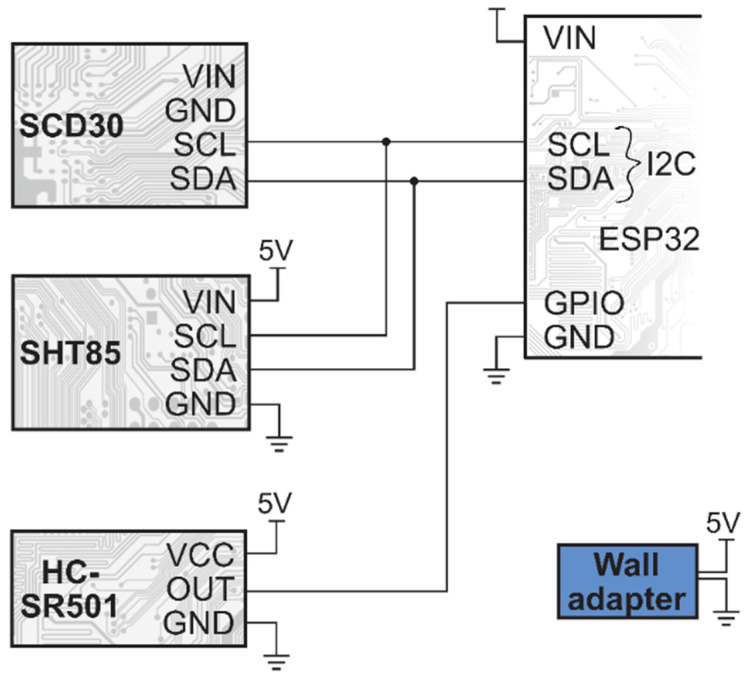
Room module schematic diagram.

The Sensirion units are connected to an I2C bus of the ESP32, and measurements are read periodically every 10 s and stored in memory along with a timestamp from the RTC. The PIR sensor is connected to a GPIO pin set to edge-triggered interrupt mode. If an interruption occurs, the timestamp of the event is stored in memory. The Wi-Fi connection is enabled once every 10 min for uploading the results to the server. Otherwise, Wi-Fi is disabled. This was required to minimise interference with motion detection. If the Wi-Fi radio operates at a high transmission power (the sensor is far from the access point), it can cause noise in the PIR sensor, which can accidentally trigger a false motion detection event. Therefore, during the (few seconds) lengths of the Wi-Fi transmission, the motion detection is disabled. The room module schematic diagram is shown in Figure 4.

#### 2.2.3. Window Measurement

The windows can be characterised by two measurements: the position of the rolling shutters (vertically) and the window’s open/tilted/closed status. To measure these characteristics, we used FM-106 reed switches (Tane Alarm, USA) [37]. This electromechanical switch consists of flexible metal contacts inside a hermetically sealed glass case. The switch is operated by applying a magnetic field (typically from an electromagnet or a permanent magnet). 

The shutter’s measurement is carried out by placing multiple reed switches on the external window frame and a permanent magnet on the bottom edge of the moving shutter, close to the external window frame. The number of switches is determined by the required resolution of the shutter’s position. In our measurements, three switches proved sufficient for an average-sized window. As the shutter is lifted or lowered, the magnet will pass the switches one by one, closing each contact for a short interval. The switches are connected to the ESP32 module’s GPIO pins with pull-up resistors. Therefore, the closing of the contact can be detected as an edge change by the microcontroller.

The position of the window sash is also measured using reed switches on the internal frame and permanent magnets on the internal side of the sash. The key to successfully distinguishing the three possible states (closed, tilted, open) is the correct placement of two switches. The first switch and magnet are placed on the bottom edge, and the contact should stay closed when the window is closed or tilted. The second switch and magnet are placed at the top, and the contacts should open when the window is tilted or fully open. The ESP32 module again detects the edge changes generated by the switches. The window’s position can always be determined using the data from these two sensors.

Providing mains power at each measured window in the apartment is problematic since the necessary cabling would annoy the tenants, since there were no wall power sockets near the measured window. Therefore, these modules are battery-powered, which requires careful hardware and software design. Changing batteries every few weeks is not just an inconvenience but also a high added cost and not an environmentally friendly solution. Currently, the system is optimised to a level that each window module sensor node can operate for approximately four months using two AA lithium batteries.

The power consumption minimisation in the hardware design is not complex in this case. The only consumers are the pull-up resistors (only when the switch is closed) and the ESP32 module itself. Choosing relatively high pull-up values and removing unnecessary extra components (LEDs, etc.) from the ESP32 module proved to be sufficient.

**Figure 5:**
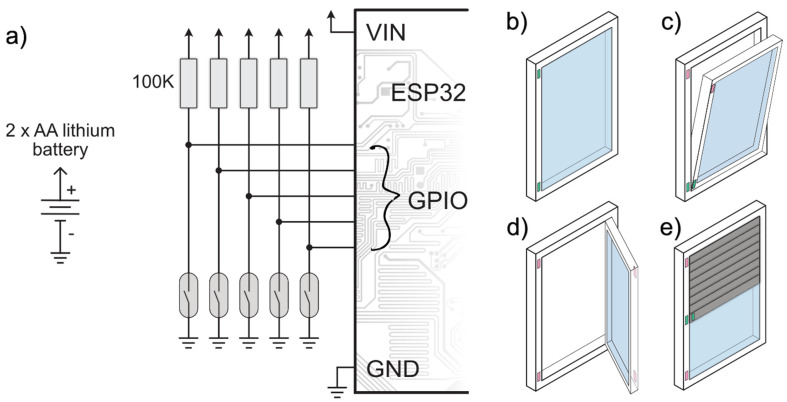
(**a**) Window module schematic diagram; (**b**) closed window from inside showing the position of reed switches and both are closed; (**c**) tilted window from inside, bottom reed switch is closed; (**d**) fully open window from inside, both reed switches are open; (**e**) position of the reed switches recording shutter position from external side, middle is closed, showing the shutter is half closed.

More effort is needed to achieve the required battery life on the software side. The ESP32 features an ultra-low power coprocessor (ULP), which can perform some simple operations while the main core is in a deep sleep. The ULP monitors the GPIO pins, where the reed switches are connected in our system, and stores the RTC timestamp if an edge change is detected. The main core only wakes up hourly to collect and upload the captured events to the server. As a result, the long-term average energy consumption of the sensor node is of the order of microwatt-hour. The schematic of the window module and the operation of the reed switches are shown in Figure 5.

#### 2.2.4. Gateway

We used a router with OpenWRT installed to provide an internet connection for the sensor nodes. It enables more customisation of the router’s configuration, e.g., using a VPN connection, logging the RSSI of the sensor nodes, etc. The router is connected to the internet using the cable connection of the apartment or an LTE USB modem. The traffic between the router and the server is encrypted using a Wireguard VPN tunnel [38]. This solution has multiple benefits: it is not strictly necessary to implement extra security measures in higher layers (e.g., encrypted MQTT), use signed binaries for firmware upgrades, etc. It makes provisioning the nodes easier, as there is no need for managing keys for each node, certificates, etc. The server and the nodes form a LAN-like network inaccessible from untrusted networks. Using this solution, the router is reachable through the VPN tunnel (e.g., for SSH) without port forwarding on the apartment’s own router.

#### 2.2.5. Server

**Figure 6:**
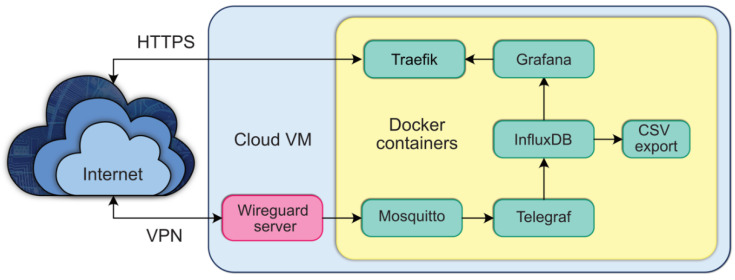
Overview of the server configuration.

The server-side components (Figure 6) are hosted on a cloud virtual machine instance, running a standard Ubuntu 22.04 Linux distribution [39]. Wireguard is installed directly and serves as the endpoint that the gateway connects. The rest of the system is deployed in Docker containers to simplify installation, updates, config, and data management. The measurements sent from the sensor nodes using the MQTT protocol are received by an MQTT broker (Eclipse Mosquitto 2.0 [40]). The persistent storage of measurements is handled by a time-series database (InfluxDB 1.8 [41]). A separate application (Telegraf 1,23 [42]) is responsible for subscribing to the MQTT topics and forwarding the incoming measurements to InfluxDB.

**Figure 7:**
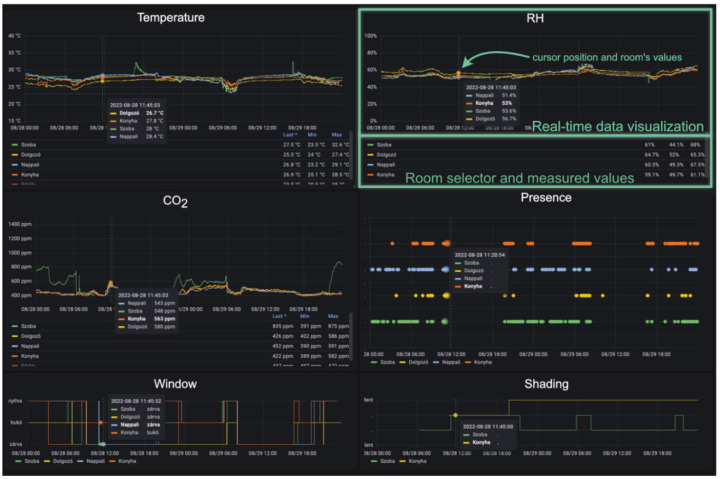
Web application visualisation example.

For visualisation, the Grafana 9.0 web application [43] is used (see Figure 7), which connects directly to the database and displays the required measurements on a custom dashboard. We use the HTTPS protocol to provide secure access to Grafana from the internet. It is handled by a dedicated reverse proxy application (Traefik 2.8 [44]) which operates the certificate generation using Let’s Encrypt [45] and forwards traffic to the Grafana container.

A custom Python [46] script runs each week to read the new measurements from the database and export them to CSV format, which is used in the later evaluation of the results.

### 2.3. Installation of the Monitoring System

**Figure 8:**
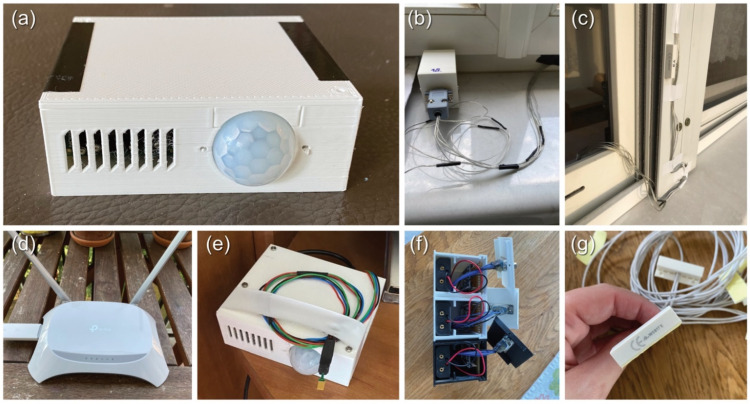
Monitoring system installation: (**a**) room module, (**b**,**c**) window sensors, (**d**) Wi-Fi module, (**e**) room module, (**f**) window module box and internal parts, (**g**) window and shutter magnetic reed switches.

The LiDAR survey provided all the data needed to build the monitoring system (see Figure 2). This way, measurements could be taken after the on-site visit, allowing the length of the cables for the windows to be planned. The system was designed and installed in a prefabricated way, with only the power sources having to be provided on-site. Double-sided adhesive tape was used to secure the position of the cables and sensors. Parts of the system are visible in Figure 8; when placing the sensors in the room, care had to be taken to ensure that they were in a relatively sheltered location, out of direct sunlight, which could affect the results. After the installation, we had a short test period to ensure that all sensors were working correctly and recording and transmitting data.

### 2.4. Evaluation of Measurement Results

The evaluation of the monitoring measurement results depends on the study’s specific purpose. In this case, we focus on temperature values and summer overheating of the apartment and highlight the importance of user habits. When processing the raw data, the first step is to review and clean the data, filter out any erroneous values, and then reduce the dataset by taking hourly averages, which can be used, e.g., in the dynamic simulations later. In this study, various statistical indicators were used to investigate and characterise the measured values, and the ODH_26_ indicator was used to describe the overheating in the apartment.

The ODH_26_ indicator, which has been used in several studies [47,48], stands for “Overheating Degree Hours above 26 °C”, which is the number of hours of temperature above 26 °C, used to determine how long and to what extent the reported overheating lasted (measured in Kh/a, i.e., Kelvin × hours/year). With a single number, we can tell the duration and extent of overheating, e.g., for houses, flats, or even rooms. In this case, the air temperature data per room are used to determine the severity of the overheating. The applied limit value of 26 °C is included as a threshold value for summer overheating in the regulations of several countries, including Hungary [8].

## 3. Results and Discussion

### 3.1. Temperature and Relative Humidity

**Figure 9:**
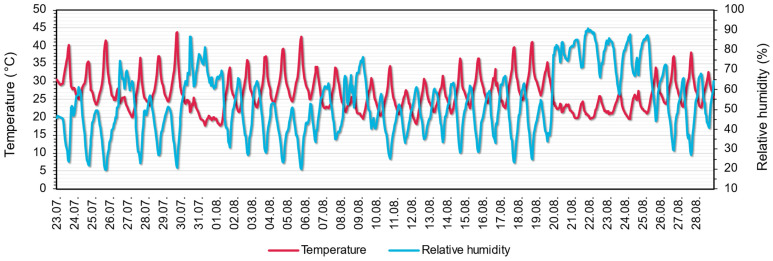
Measured external temperature and relative humidity.

Figure 9 shows the external measured temperature and relative humidity values. The daily temperature and relative humidity fluctuations are clearly visible from the measured dataset. The maximum value measured on the balcony was almost 44 °C, while the average outside temperature was 26.5 °C, with a 4.75 °C deviation and a median value of 27.7 °C. Although no precipitation was measured, the high relative humidity values (around 80–90%) identify the precipitation and the subsequent cooling around 20 August, accompanied by a smaller temperature fluctuation than the dry days.

**Figure 10:**
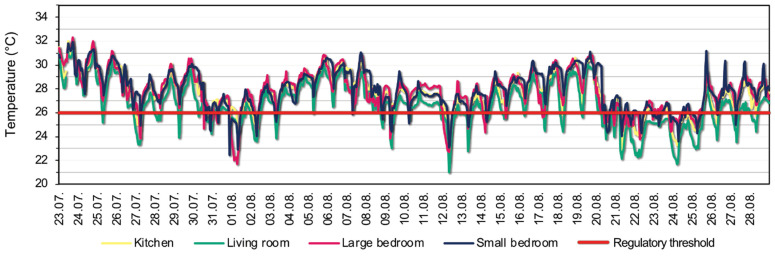
Measured temperature values within the apartment, including the 26 °C regulatory threshold temperature.

**Figure 11:**
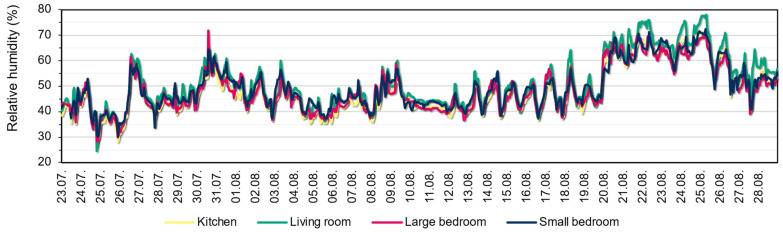
Measured relative humidity values within the apartment.

Figure 10 and Figure 11 show the measured temperature and relative humidity values in the rooms between 23 July and 28 August 2022, respectively. It can be seen that the measured values are similar from room to room, with slightly larger differences in temperature between the living room and the small bedroom. There is no significant difference in relative humidity between rooms. It is visible that the average daily temperatures are above the 26 °C regulatory threshold, except on rainy days. The temperatures during day and night show large differences, but there were multiple days where the temperature was above the threshold even at night. Around the end of the measured month, the living room tends to produce higher relative humidity during the day for the multiple rainy days compared to the other rooms. It correlates with the lower temperature in the living room on rainy days.

The main statistical indicators are shown in Table 1. The maximum temperatures were 32.04 °C in the kitchen, 31.52 °C in the living room, 31.94 °C in the small bedroom, and 32.31 °C in the large bedroom. These values are much higher than the often-considered thermal comfort limit of 26 °C. During the measured period, there were also cooler periods when the internal temperature reached 20–21 °C, when, with adequate ventilation, the external temperature effectively cooled the dwelling. However, the average room temperature remained above 26 °C. The hottest room (27.9 °C) was the small bedroom in the analysed period, but the large bedroom and living room also had a high average temperature (above 27 °C), with a 1.6–1.8 °C deviation in general. The relative humidity averaged 50% in the rooms during the measurement, ranging from 24.59% to 77.93%.

**Figure 12:**
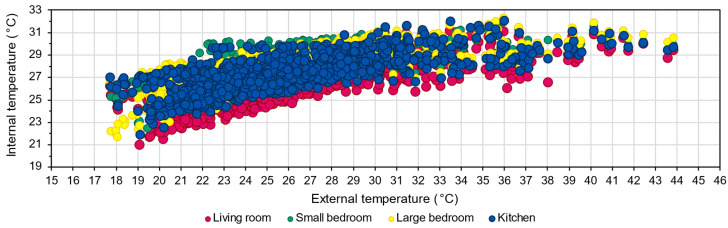
Internal room temperatures as the function of the external temperature within the measurement period.

Figure 12 shows the internal temperatures in the function of the external measurement; this indicates that the temperature in the living room is significantly lower than in the other rooms. It also shows that the internal temperature peaks are mainly concentrated in the external temperature range of 33–36 °C.

### 3.2. Overheating Measures

We can analyse specific threshold values for the indoor space when looking at overheating. In general, 26 °C is considered a limit for overheating, so this value is considered in the analysis. In addition, two indoor limits of 28 °C and 30 °C, as high extremes, were observed in the dwelling.

**Figure 13:**
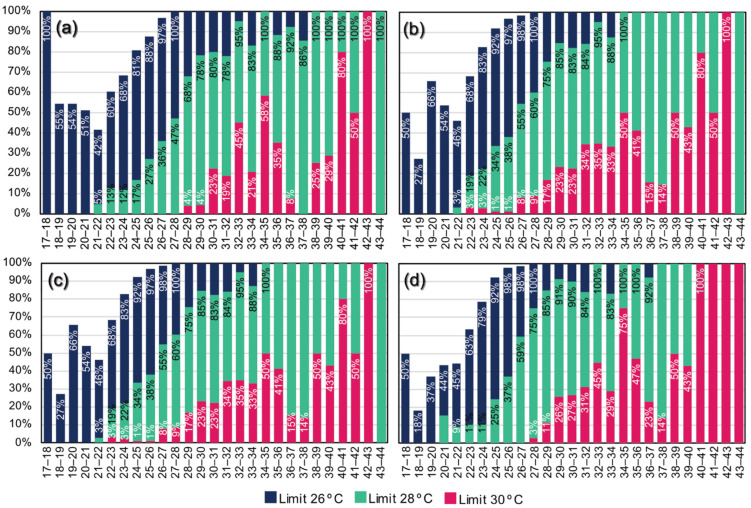
Internal temperature limit exceedance according to external temperature; y-axis: the probability of exceeding a certain limit based on the measurements; x-axis: external temperature. (**a**) Kitchen, (**b**) small bedroom, (**c**) living room, (**d**) large bedroom.

Figure 13 shows the number of times a given room exceeded these limits at a given outside temperature range. Hence, it shows the relative overheating of rooms for different outdoor temperature ranges. Based on the results of the summer month in question, there is at least a 50% chance that the indoor temperature will rise above 26 °C even if there are small cooler periods (17–18 °C). Overall, the living room and kitchen are less exposed to the significantly elevated internal overheating of 28 °C to 30 °C, partly due to their orientation and partly due to user habits (less use, better ventilation). However, the two bedrooms often exceed the 30 °C limit. Above an outside temperature of 39–40 °C, there is a 43% chance that the inside temperature will be above 30 °C. At higher temperatures, especially in the large bedroom, the temperature will always rise above 30 °C. It is an exceptionally high risk, as these rooms are usually used when residents are at home. Based on these measurements, by analysing longer-term data series, we can conclude how the building will warm up based on weather forecasts (with similar usage patterns). 

The previously presented ODH_26_ indicator is an efficient way to characterise and compare the summer overheating of dwellings and rooms (see Table 2). The indicator value in the two bedrooms is the highest at over 1700 Kh, while the lowest in the living room is around 1000 Kh. It can therefore be seen that the overheating in different parts of the dwelling is much more variable, which can be explained partly by the orientation and design of the dwelling and partly by differences in use.

**Figure 14:**
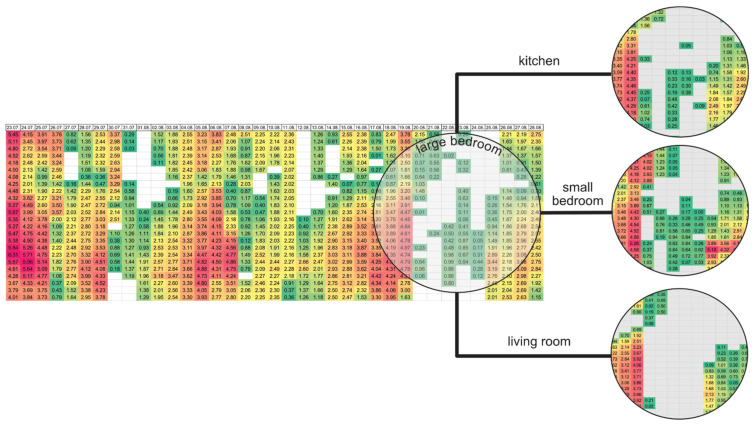
ODH_26_ values by hours in the large bedroom, the difference between the rooms between 18 and 28 August 2022.

In addition to the previous measure, Figure 14 shows the number of hours in the period when the internal temperature was above 26 °C in the rooms. The colour of the cells shows the extent to which this limit was exceeded. It suggests that there were three significant warming periods during the study period: one at the beginning of the measurement (23–26 July), one at the beginning of August (5–7 August), and one in the middle of August (16–19 August). According to data from the Hungarian Meteorological Service (OMSZ), during this period, there were eight heatwave days during this period when the mean outdoor temperature exceeded 25 °C. Their distribution over time is similar to that observed for internal temperatures. Comparing this visualisation in the rooms studied, the differences in the last period are particularly noticeable. The living room had barely any hours above 26 °C during this period; the kitchen is relatively similar to the living room, but in the small bedroom, the number of hours of elevated temperature is higher, and there are warmer hours than in the large bedroom.

### 3.3. User Behaviour

**Figure 15:**
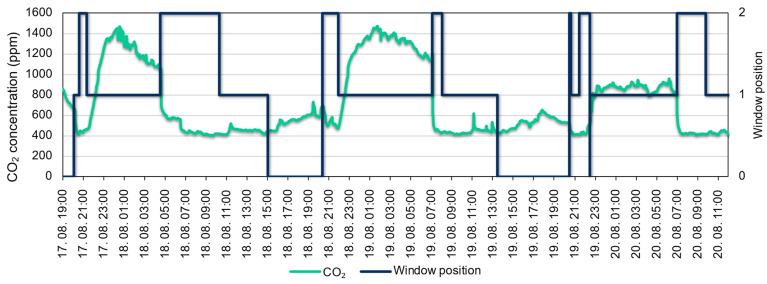
CO_2_ values in the small bedroom and window position (“0”—closed, “1”—tilted, “2”—fully open).

A period in mid-August (17–20 August 2022) was chosen to study user behaviour and habits. Figure 15 shows the CO_2_ concentration measured in the small bedroom during the period under study and the measured window opening. For window opening, “0” means the window is closed, “1” means the window is tilted, and “2” means the window is fully open. For the small bedroom, the internal door is usually closed at night. According to the diagram, on the evening of 17.08, the window was operated in tilted mode, during which time the CO_2_ concentration in the room accumulated (up to 1400 ppm). The window was fully opened in the early morning hours, around 04:30 a.m., so it was possible to ventilate and reduce the CO_2_ concentration relatively quickly and effectively. Other similar ventilation periods can also be observed in the figure.

**Figure 16:**
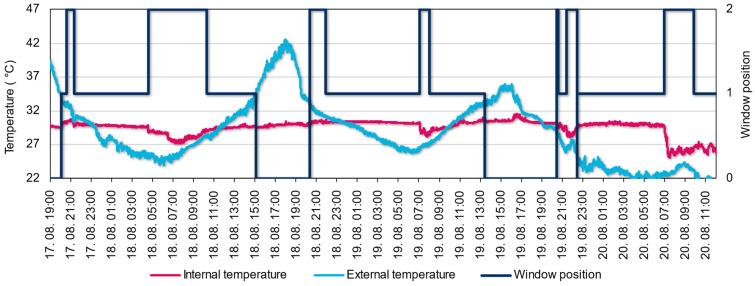
External and internal temperatures in small bedroom and window position (“0”—closed, “1”—tilted, “2”—fully open).

One of the reasons for the increased ventilation at dawn could be the extremely high internal temperature, which was over 29.7 °C in the bedroom during this period. Figure 16 shows that ventilation was much more efficient after the window was fully opened, with a drop of more than 2 °C in the internal temperature in a short period. Another example of ventilation efficiency is the ventilation strategy on 20 August. External measurements show that the outside temperature dropped below 22 °C during this time. As long as the window was only open for a short time and tilted mode, the ventilation could not be efficient; as soon as the occupant opened the window fully, the internal temperature dropped drastically by 5 °C. Measurements and analyses such as these will help to develop more efficient ventilation strategies in the future, as well as to calibrate dynamic simulations. The measurements will allow us to analyse the air exchange in the study area and develop occupant profiles.

## 4. Conclusions

We developed a low-cost wireless monitoring sensor system that can collect temperature, relative humidity, CO_2_, and user behaviour data such as window opening and external shutter position. The designed monitoring system was constructed and installed in an apartment located in a large concrete panel building to measure the temperature and user behaviour, since this type of building has proven to be one of the most vulnerable to heatwaves. The possibility of measuring window sash and shutter position to monitor the window tilting or opening and the shutter operation is among the novel capabilities of our developed sensor system. We developed a web application to visualise real-time measurement data, and its dashboard can be freely changed and combined, which usually cannot be achieved with commercial systems.

We used a LiDAR-based survey to obtain the building’s floor plan and internal layout. The 2020 iPad Pro’s LiDAR sensor proved to be a fast and adequate solution for the task, providing acceptable accuracy for the drafting.

During the analysed summer month following the installation of the wireless monitoring system, it was found that although residents reported that the temperature in their homes was tolerable during the summer, there were frequent cases of temperatures above 30 °C in the rooms, well above the accepted summer comfort limit of 26 °C. Bedrooms were found to be the hottest rooms, both during the day and at night, with more than a 40% difference in the values of the ODH_26_ indicator.

Examining ventilation habits shows that if the outside temperature is adequate, effective ventilation at night can significantly reduce the internal temperature. As in the case of the apartment under study, it is also necessary to consider the CO_2_ concentration when assessing indoor comfort, which can rise slightly or stagnate even with a tilted window but can only be effectively reduced when the window is fully open.

The research and the monitoring system that has been developed will contribute to a deeper understanding of how our buildings work and how climate change is increasing the frequency and severity of overheating in buildings. The recorded measurements can be used to perform various analyses or for further dynamic simulation studies. We can create user profiles with an extensive dataset to make our simulations more accurate. The elements of the measurement system can usually be reused later, i.e., it can be installed at a new measurement site with minor modifications after the measurement is finished. Our study will help to develop more efficient ventilation strategies in the future, as well as to calibrate dynamic simulations.

The measurement of user patterns was limited to the external building envelope (windows and shutters). For further studies, it may be worth measuring the movement of internal doors, as these can impact the internal air quality. Long-term measurements will continue during the winter for a whole year, during which time changes in user habits according to the seasons may be observed.

## Figures and Tables

**Table 1:** Statistics of the measured temperature and relative humidity (K—kitchen, LR—living room, SB—small bedroom, LB—large bedroom, E—external balcony).

	K	LR	SB	LB	E	K	LR	SB	LB	E
	Temperature (°C)					Relative Humidity (%)				
Min.	21.91	20.98	22.45	21.70	17.73	26.12	24.59	30.06	28.22	19.57
Max.	32.04	31.52	31.94	32.31	43.84	71.14	77.93	72.49	71.91	90.63
Mean	27.57	26.72	27.90	27.77	26.51	47.97	50.93	49.46	48.21	52.99
Std. Dev.	1.64	1.81	1.64	1.73	4.75	9.19	10.08	8.56	8.70	16.46
Median	27.58	26.71	27.85	27.93	25.72	45.78	48.02	47.42	46.06	50.88

**Table 2:** ODH_26_ values during the measurement period.

Room	ODH_26_ Values
Kitchen	1543 Kh
Living room	1013 Kh
Small bedroom	1765 Kh
Large bedroom	1717 Kh

## Data Availability

Not applicable.
